# Degradation of soil quality by the waste leachate in a Mediterranean semi-arid ecosystem

**DOI:** 10.1038/s41598-021-90699-1

**Published:** 2021-05-31

**Authors:** Sh. Yeilagi, Salar Rezapour, F. Asadzadeh

**Affiliations:** Soil Science Department, Urmia University, P.O. Box 165, Urmia, 57134 Islamic Republic of Iran

**Keywords:** Environmental sciences, Natural hazards, Health occupations

## Abstract

The assessment of soil quality indices in waste leachate-affected soils is vital to understand the threats of land quality degradation and how to control it. In this respect, a study was conducted on the effects of uncontrolled landfill leachate on soil quality index (SQI) in calcareous agricultural lands using 28 soil variables. Using the total data set (TDS) and minimum data set (MDS) approaches, the SQI was compared between leachate-affected soils (LAS) and control soils by the integrated quality index (IQI) and nemoro quality index (NQI) methods. The results revealed that LAS were significantly enriched by soil salinity-sodicity indices including electrical conductivity (EC), sodium adsorption ratio (SAR), and exchangeable sodium percentage (ESP), fertility indices including total N, available P and K, organic carbon, and cation exchange capacity (CEC), exchangeable cations (Ca, Mg, K, and Na), the available and total fractions of heavy metals (Zn, Cu, Cd, Pb, Ni). After the leachate got its way into the soil, the values of IQI and NQI were dropped ranging 5–16% and 6.5–13% for the TDS approach and 5–15.2% and 7.5–12.2 for the MDS approach, respectively. Clearly, the data showed that soil quality degradation was encouraged and stimulated by the leachate. Among the different models of SQI applied in the present study, IQI determined by MDS was the optimal model to estimate soil quality and predict crop yields given the analysis of the correlations among the SQI models, the correlations between the SQI models and wheat yield, and sensitivity index values.

## Introduction

Presently, one of the most important environmental challenges of the world posed by rapid population growth and urbanization is the generation of a high volume of municipal solid waste (MSW) that can seriously threaten the health of soil–water–plant–human systems. Based on the World Bank Group, the global generation of MSW amounted to 1.3 billion tons year^−1^ in 2018, expected to reach approximately 2.2 billion tons by 2025^1^. This huge volume of waste can pose a great challenge mainly in low- and middle-income countries and developing nations. In general, MSW is generated from various sources, e.g., industrial, agricultural, service, commercial, and household activities or everyday items discarded by the public. The quality and composition of MSW vary by activity type and involves plastic, food waste, inorganic salts, glass, paper, building and electronic wastes, metals, and organic fractions^2^. As a result of biodegradation and various physical, chemical, and biological reactions occurring in MSW, leachate is produced as a by-product. Leachate is a dark brown liquid with a stinking smell that is excreted inside MSW and contains soluble and suspended material. The leachate composition contains inorganic and soluble organic and inorganic compounds, nutrients, suspended particles, heavy metals, and many hazardous chemicals, causing significant damage to both natural and agricultural ecosystems when released in an untreated and uncontrolled manner^3,4^. The rate of leachate production, its volume, and its properties depend on various factors, e.g., the composition of waste material, its particle size, degree of waste compaction, waste moisture and temperature, the amount of rainfall, and the quantity and quality of biochemical that occur in the degradation stages of the MSW^2,4^.

Several studies have found the negative effects of waste leachate on soil quality due to the presence of high content of nutrients, heavy metals, and soluble salts in the leachate^3,4^. For example, a range of approximately 1000–3000 mg L^−1^ for ammoniacal nitrogen was reported in leachates from different parts of the world^5^ and ranges of 0.1–40 mg kg^−1^, 20–1500 mg kg^−1^, and 15–1300 mg kg^−1^ were found for Cd, Cr, and Ni of leachates of the US, respectively^6^. The upper range of these concentrations is far beyond their acceptable ranges. However, few studies have investigated the impacts of leachate on the degradation of soil quality.

The scientific and systematic evaluation of leachates of MSW-affected soils can be a very important approach to establishing a proper management strategy to prevent soil degradation and alleviate the threats posed by the expansion of waste. In this context, the development of the Soil Quality Index (SQI) may be a useful tool to help improve the management of waste soils^8,16^. The term soil quality (SQ) is described as a component of a large ecosystem that supports plant growth and productivity, regulates water flows, maintains environmental quality, and promotes animal and human health^6^. Both qualitative and quantitative soil quality factors can be involved in soil quality assessment. Qualitative SQ assessment has been developed mainly through SQ cards, SQ test kits, and similar items^7^. The current methods of quantitative SQ assessment are soil quality indices (SQI), multiple-variable indicator kriging, the methods based on geographic information system (GIS) data, multivariate geostatic methods, and methods based on a combination of GIS data and fuzzy mathematics^8,9^. Among these methods, SQI has been widely and commonly applied in several works at different scales, locations, and ecosystems, e.g., Biswas et al.^10^ and Li et al.^11^ in Asia, Askari and Holden^12^ and Santos-Francés et al.^13^ in Europe, and Mukherjee and Lal^14^ and Sione et al.^15^ in America. Simplicity, practicability, and quantitative flexibility are the most important reasons for the common use of the SQI method^8^.

Although, valuable research works have addressed human-induced soil quality degradation in the past few decades, the quantity of SQIs in the croplands affected by uncontrolled leachate has rarely been assessed. Besides, soil heavy metals have been used in the current research to calculate the quality index, while it has not been carried out in previous studies. Heavy metals have been specified as an important factor affecting soil quality and sustainability^6^. These metals are a ubiquitous environmental pollutant which are non-biodegradable, can reduce capable soil resources, and have adverse effects on plant growth and yield^2,16^.

The present study assessed the effects of landfill leachate on SQI in cropland in northwest Iran, where dumping a large amount of MSW generates a huge amount of leachate flowing onto cropland. Understanding and estimating the quality of leachate-impacted soils can establish an opportunity to judge the sustainability of land management and land-use systems^16^. Therefore, the objectives of this study were (a) to compare changes in selected soil attributes between leachate-affected soils and the adjoining unaffected soils and (b) to evaluate the effects of landfill leachate on SQI using the indicator selection methods (total data set and minimum data set) and different SQI models.

## Material and methods

### Site characteristics and soil sampling

**Figure 1:**
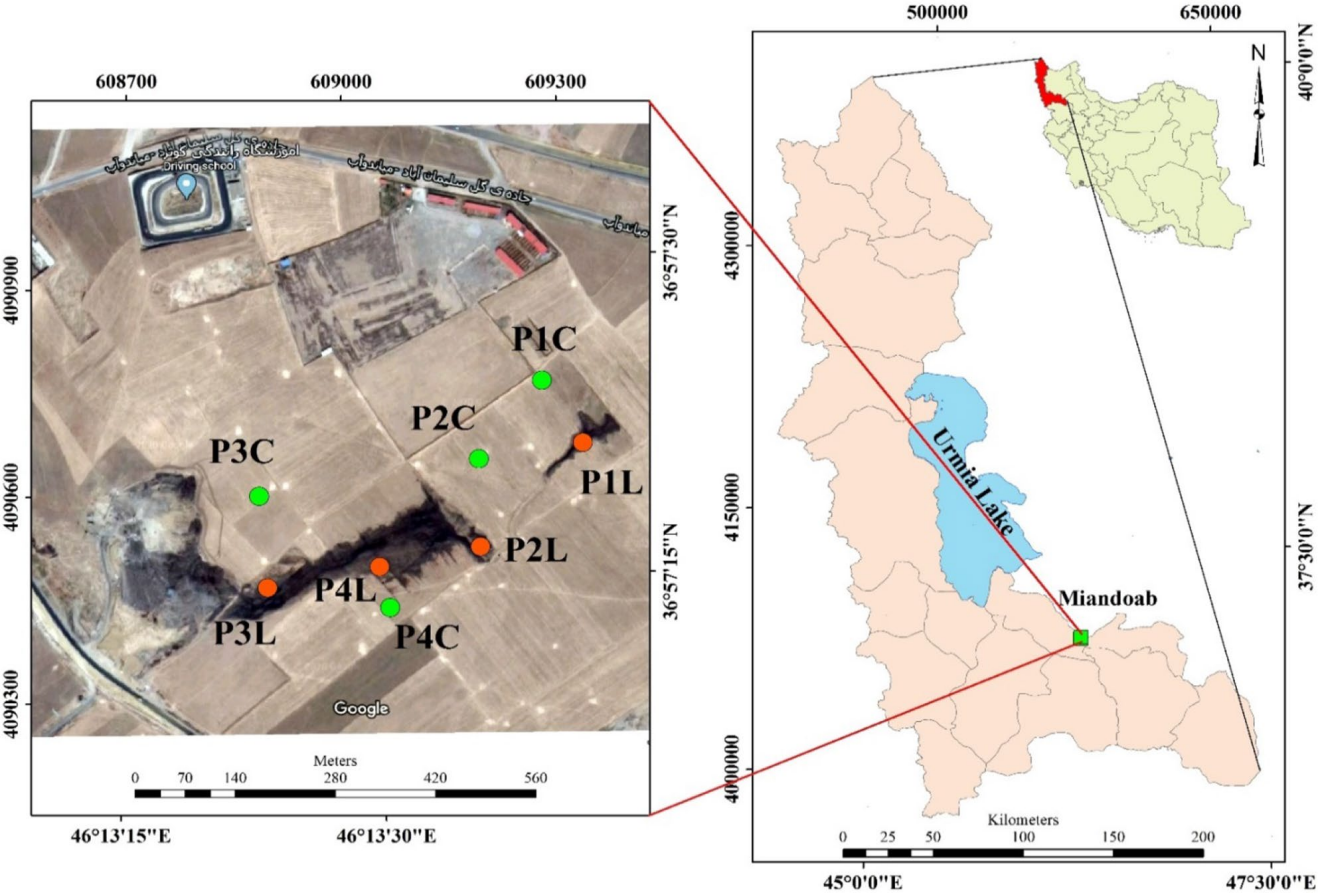
Location of the study area ( (P1L, …, the soil under influence of leachate; P1C,… the adjacent control soils) (https://www.qgis.org/ and https://www.google.com/earth/).

The study was carried out in an uncontrolled landfill site located approximately 20 km from Miandoab City in Western Azerbaijan province, north-western Iran (Fig. 1). The site is surrounded by agricultural fields where waste is buried in an uncontrolled open system and without any engineering operations. Wastes on this site are composed of the municipal solid and liquid wastes generated by commercial activities, hospitals, urban municipal, energy generation residues, and industries in Miandoab County. It is estimated that the site has received over 50 tons of waste every day for over 15 years where it is usually burned every week after it reaches a height of 10 to 15 m, resulting in the generation of dark and stinking leachate. The leachate then flows into the cropland around the site as there is no barrier system or leachate collection system in the studied landfill. This has provoked widespread discontent and protests by local farmers and residents of the area. Chemical composition of the investigated leachate is presented in Table S1.

The region has a semi-arid climate with average annual precipitation and temperature of 320 mm and 13 °C, respectively. The major crop cultivated in the region is rain-fed wheat (*Triticum aestivum *L.) growing from November to July, i.e. a growth period of about 220 days. Over three decades, conventional cultivation methods have been using tillage and fertilization. Every year, 150–200 kg ha^−1^ of urea and 100–150 kg ha^−1^ of superphosphate are applied. Superphosphate is soil-incorporated before sowing as a basal treatment, but urea is applied quantitative in two splits, i.e., two-thirds is used as a basal treatment with soil incorporation and the remaining is top-dressed during tillering and stem elongation stages of wheat growth. In the fieldwork, eight soil profiles—four from the leachate-affected soils and four from the control soils—were dug, described, and sampled along a transect at four experimental sites. Due to the similar slope, drainage conditions, parent materials, and soil types, two paired soil profiles (including leachate-affected soil and the adjacent control soil) were specified at each experimental site. A central point was selected for each soil profile, and composite soil samples were taken from four directions including north, south, west, and east of the composite soil samples. Every composite sample was made of three sub-samples. Additionally, the soil sampling process was carried out by a spade from the plow layer (at a depth of 0–0.3 m) within a radius of 5–10 m from the central point. All the soil profiles had a high level of calcium carbonate (10–31%), so the major soil group was Calcisols based on the WRB system^17^. Soil samples were air-dried, grounded, sieved through a 2-mm sieve, and kept in polythene bags for further analysis.

### Analysis of soil properties

Soil pH, electrical conductivity (EC), soil organic carbon (SOC), cation exchange capacity (CEC), and calcium carbonate equivalent CaCO_3_ (CCE) were determined by procedures described by Sparks et al.^18^. Total N and available P were estimated using the Kjeldahl approach^19^ and spectrophotometry methods^20^. Soluble and exchangeable cations were measured by the method of saturation extract and 1 N NH_4_OAc, respectively^21^. Sodium absorption ratio (SAR) was computed using the concentration of solution Na, Ca, and Mg, and exchangeable sodium percentage (ESP) was computed using exchangeable Na and CEC values^22^.

Available and total fractions of Zn, Cu, Cd, Pb, and Ni were derived by diethylene-triamine pentaacetic acid (DTPA)^23^ and concentrated nitric acid^24^, respectively. Then, the concentration of the metal was measured by an atomic absorption spectrophotometer (Shimadzu AA-6300).

### Soil quality index (SQI)

The study applied both total data set (TDS) and minimum data set (MDS) approaches to assess SQI. A total of 28 soil attributes, i.e. pH, SAR, EC, ESP, CCE, ACC (active calcium carbonate), OM (organic matter), CEC, Total N, Available P, soil ionic composition (Cl^−1^, HCO_3_^–1^, Ca^2+^, Mg^2+^, K^+^, Na^+^), bioavailable Fe and Mn, and heavy metals (Zn, Cu, Cd, Pb, Ni), were identified and considered for the SQI development using TDS. In this regard, all measurable and accessible soil data were indeed used since the TDS approach is capable of producing a comprehensive outcome in evaluating the SQI. In the MDS cause, a principal component analysis (PCA) was carried out on the TDS to reduce the dimensionality of the data and identify the most important variables to be included in the MDS. For each principal component (PC), soil variables with eigenvalue ≥ 1, which explained at least 5% of the variance in the data, were selected. The variables with a high absolute loading value obtaining weighted loading amounts within 10% of the highest weighted factor were considered for the MDS. When more than one variable was presented in any PC, their correlation was analyzed to retain only one in the MDS^8^. Otherwise, each the indicator with the highest loaded was kept in the MDS.

The data were transformed into dimensionless scores using the standard scoring functions (SSF). Based on the sensitivity of functions in estimating soil quality, three types of SSF involved "the lower, the better", "the upper, the better", and the optimum range. In the study, the "more range" function was used for the soil fertility indices (e.g., OM, CEC, total N, available P, and K); the "low range" function for soil sodicity indices (e.g., SAR, ESP, exchangeable Na) and heavy metals; and the "optimum range" function for pH (the threshold value of 6.5–7.5), EC (the threshold value of 0.2–2 dS m^−1^), and the bioavailable fraction of Zn (the threshold value of 0.6–10 mg kg^−1^) and Cu (the threshold value of 0.2–5 mg kg^−1^)^8,10,19,52^.

The following scoring curves were used as "more or positive range" (Eq. (1)) or "less or negative rage" (Eq. (2)) functions^10^:

1 $$SL=\frac{Xi-{X}_{min}}{{X}_{max}-{X}_{min}}$$

2 $$SL=\frac{{X}_{max}-Xi}{{X}_{max}-{X}_{min}}$$

where *SL* = the linear score, *Xi* = the soil variable content, *X*_*min*_ = the minimum content of soil variable, and X_*max*_ = the maximum content of soil variable.

Further, the SQI was computed using two models including the Integrated Quality Index (IQI)^25^ and Nemoro Quality Index (NQI)^26^ for the TDS and MDS approaches as expressed in Eqs. (3) and (4), respectively:

3 $$IQI=\sum_{i=1}^{n}{W}_{i}\times {S}_{i}$$

where S_i_ is the variable score, n is the number of soil variable, and W_i_ is the weighting value of soil variable. The weight values were equal to the ratio of the communality of each variable to the sum communalities of all variables.

4 $$\mathrm{NQI}=\sqrt{\frac{{P}^{2}ave+{P}^{2}min}{2}} \times \frac{n-1}{n}$$

where *P*^2^*ave* and *P*^2^*min* are the average and minimum scores of the selected variables, respectively.

In the current study, four different SQIs were assessed using the IQI and NQI methods, the TDS and MDS approaches, and linear scoring methods. Each SQI was divided into five categories involving very high (I), high (II), moderate (III), low (IV), and very low (V) (Table 1)^15^.

**Table 1:** Categorization of soil quality grades using different methods.

SQI senario	Soil quality grade				
	I (very high)	II (high)	III (moderate)	IV (low)	V (very low)
IQI-T	> 0.64	0.58–0.64	0.52–0.58	0.46–0.52	< 0.46
NQI-T	> 0.43	0.40–0.43	0.37–0.40	0.34–0.37	< 0.34
IQI-M	> 0.66	0.58–0.62	0.54–0.58	0.51–0.54	< 0.51
NQI-M	> 0.37	0.34–0.37	0.31–0.34	0.28–0.31	< 0.28

IQI: Integrated Quality Index; NQI: Nemoro Quality Index; T: total data set; M: minimum data set.

To compare and evaluate the accuracy of different SQI models, the sensitivity index and correlation analysis were performed among the SQIs as well as between different SQI methods and wheat grain yield. The sensitivity index was calculated by Eq. (5) as follows^27^.

5 $$\mathrm{SI }= \frac{{SQI}_{(max)}}{{SQI}_{(min)}}$$

where *SQI*_*(max)*_ and *SQI*_*(min)*_ are the maximum and minimum of each SQI model, respectively. The higher SI values were assumed to represent more sensitivity potential of the SQI models, impacting both natural and anthropogenic processes^27^.

All statistical analyses (i.e., standard deviations and correlations) were performed by the SPSS 19 software package (SPSS INC., Chicago, USA). The means of different soil variables and different SQI scenarios were compared between the leachage-affected soils and the control soil using a paired t-test.

## Results and discussion

### Indices of soil salinity-sodicity and fertility

Table 2 shows the effects of uncontrolled landfill leachate on the selected indices of soil salinity-sodicity and fertility. The pH of the leachate-affected soils (LAS) was slightly lower (7.3 to 7.5) than the control soils (7.5 to 7.8). A significant rise of 78 (site 1) to 114% (site 3) occurred in soil EC after the leachate entered the soil probably due to the presence of high levels of cations and anions as well as total dissolved solids in the leachate itself (Table 1). These results are consistent with those reported by Nareen et al.^3^ and Somani et al.^28^. The values of SAR and ESP increased significantly in the LAS compared with the control soil in all soil sites. The leachate inflow into the soil caused a rise of 42–67% and 35–71% in SAR and ESP, respectively, which may have aggravated soil quality degradation, particularly the degradation of soil physical properties (e.g. slaking, swelling, and dispersion of clay fraction). The leachate in the soils increased their fertility indices (e.g., OM, CEC, total N, available P, and K) in a range of 15–102%, implying the positive and improving impact on soil quality. The concentrations of exchangeable cations were in the order of Ca > Mg > Na > K and Ca > Mg > K > Na in the LAS and control soils, respectively. The incorporation of leachate into soil produced a slight increase in exchangeable Ca and Mg, whereas exchangeable K and Na were significantly (P ≤ 0.05) enhanced by leachate.

**Table 2:** Comparison of soil variables values (mean ± SD) between LAS and adjacent control soil (n = 60).

Soil attributes	Site 1		Site 2	
	LAS	Control soil	LAS	Control soil
pH	7.5 ± 0.3^a^	7.7 ± 0.1^a^	7.4 ± 0.28^a^	7.6 ± 0.13^a^
EC (dS m^−1^)	3.5 ± 1.1^a^	1.87 ± 0.4^b^	3.79 ± 1.6^a^	1.87 ± 0.31^b^
SAR	5.92 ± 1.1^a^	3.84 ± 0.42^b^	4.52 ± 0.95^a^	2.83 ± 0.4^b^
ESP (%)	5.84 ± 1.2^a^	4.01 ± 0.38^b^	7.3 ± 1.41^a^	4.54 ± 0.92^b^
OM (g kg^−1^)	18.7 ± 0.6^a^	16.2 ± 0.2^b^	21.8 ± 0.5^a^	17.5 ± 0.3^b^
CEC (cmol kg^−1^)	21.9 ± 3.3^a^	18.8 ± 1.9^b^	24.0 ± 2.8^a^	20.1 ± 1.1^b^
CCE (g kg^−1^)	301 ± 10.6^a^	280 ± 7.7^a^	320 ± 10.3^a^	295 ± 8.7^a^
Total N (%)	0.25 ± 0.0.05^a^	0.2 ± 0.01^b^	0.33 ± 0.02^a^	0.21 ± 0.01^b^
Available P (mg kg^−1^)	12.8 ± 0.4^a^	8.86 ± 0.19^b^	11.9 ± 0.6^a^	9.09 ± 0.27^b^
Available K (mg kg^−1^)	440 ± 25.4^a^	337.6 ± 19.9^b^	449.5 ± 37.5^a^	379.7 ± 30.2^b^
Exchangeable Ca (cmol_c_ kg^−1^)	15.8 ± 0.61^a^	13.3 ± 0.52^b^	14.9 ± 0.84^a^	13.1 ± 0.75^b^
Exchangeable Mg (cmol_c_ kg^−1^)	4.6 ± 0.32^a^	4.0 ± 0.21^b^	4.2 ± 0.28^a^	3.7 ± 0.24^b^
Exchangeable K (cmol_c_ kg^−1^)	1.23 ± 0.04^a^	0.91 ± 0.02^b^	1.2 ± 0.0.8^a^	0.90 ± 0.02^b^
Exchangeable Na (cmol_c_ kg^−1^)	0.55 ± 0.06^a^	0.36 ± 0.03^b^	0.62 ± 0.34^a^	0.38 ± 0.17^a^
Zn-DTPA (mg kg^−1^)	14.5 ± 2.5^a^	2.4 ± 0.11^b^	14.3 ± 2.7^a^	1.2 ± 0.2^b^
Cu- DTPA (mg kg^−1^)	7.2 ± 1.4^a^	1.3 ± 0.3^b^	6.4 ± 0.6^a^	1.3 ± 0.12^b^
Cd-DTPA (mg kg^−1^)	0.65 ± 0.23^a^	0.4 ± 0.09^b^	0.59 ± 0.1^a^	0.34 ± 0.03^b^
Pb DTPA (mg kg^−1^)	3.1 ± 0.36^a^	1.1 ± 0.26^b^	3.3 ± 0.31^a^	1.1 ± 0.21^b^
Ni-DTPA (mg kg^−1^)	2.2 ± 0.25^a^	0.9 ± 0.05^b^	2.7 ± 0.2^a^	1.4 ± 0.04^b^
Total Zn (mg kg^−1^)	162.9 ± 8.2^a^	63.8 ± 1.8^b^	161.9 ± 6.3^a^	61.3 ± 1.5^b^
Total Cu (mg kg^−1^)	58.3 ± 2.1^a^	46.2 ± 1.6^b^	55.5 ± 1.8^a^	45.1 ± 1.3^b^
Total Cd (mg kg^−1^)	5.2 ± 1.2^a^	1.8 ± 0.5^b^	5.1 ± 1.1^a^	1.7 ± 0.3^b^
Total Pb (mg kg^−1^)	63.2 ± 2.3^a^	38.9 ± 0.85^b^	77.8 ± 1.9^a^	38.8 ± 0.71^b^
Total Ni (mg kg^−1^)	57.5 ± 2.5^a^	30.5 ± 0.98^b^	59.1 ± 1.7^a^	31.2 ± 0.76^b^

Soil attributes	Site 3		Site 4	
	LAS	Control soil	LAS	Control soil
pH	7.3 ± 0.14^a^	7.5 ± 0.1^a^	7.5 ± 0.2^a^	7.8 ± 0.14^a^
EC (dS m^−1^)	5.98 ± 2.1^a^	2.8 ± 0.8^b^	3.4 ± 1.2^a^	1.65 ± 0.5^b^
SAR	5.94 ± 0.91^a^	3.91 ± 0.53^b^	6.6 ± 0.98^a^	3.95 ± 0.44^b^
ESP(%)	4.84 ± 1.03^a^	3.03 ± 0.31^b^	6.65 ± 0.81^a^	3.9 ± 0.3^b^
OM (g kg^−1^)	19.0 ± 0.3^a^	15.7 ± 0.2^b^	22.7 ± 0.43^a^	18.5 ± 0.24^b^
CEC (cmol kg^−1^)	18.4 ± 2.8^a^	15.6 ± 1.1^b^	24.6 ± 3.4^a^	20.63 ± 2.1^b^
CCE (g kg^−1^)	282 ± 10.3^a^	263 ± 8.7^b^	310 ± 8.3^a^	291 ± 7.6^a^
Total N (%)	0.21 ± 0.02^a^	0.15 ± 0.01^b^	0.23 ± 0.03^a^	0.18 ± 0.01^b^
Available P (mg kg^−1^)	10.45 ± 0.67^a^	8.19 ± 0.27^b^	12.17 ± 0.54^a^	10.21 ± 0.34^b^
Available K (mg kg^−1^)	420 ± 37.5^a^	340 ± 30.3^b^	417 ± 29.8^a^	342 ± 25.7^b^
Exchangeable Ca (cmol_c_ kg^−1^)	13.4 ± 0.84^a^	11.9 ± 0.73^b^	15.5 ± 0.61^a^	14.0 ± 0.54^a^
Exchangeable Mg (cmol_c_ kg^−1^)	3.7 ± 0.2^a^	3.3 ± 0.1^a^	4.3 ± 0.3^a^	3.9 ± 0.2^a^
Exchangeable K (cmol_c_ kg^−1^)	1.01 ± 0.06^a^	0.86 ± 0.04^b^	1.2 ± 0.11^a^	0.97 ± 0.03^b^
Exchangeable Na (cmol_c_ kg^−1^)	0.68 ± 0.2^a^	0.38 ± 0.1^b^	0.49 ± 0.09^a^	0.26 ± 0.08^b^
Zn-DTPA (mg kg^−1^)	17.8 ± 1.2^a^	1.5 ± 0.21^b^	23.8 ± 1.4^a^	0.9 ± 0.1^b^
Cu- DTPA (mg kg^−1^)	5.3 ± 0.78^a^	1.2 ± 0.2^b^	6.7 ± 0.57^a^	1.1 ± 0.1^b^
Cd-DTPA (mg kg^−1^)	0.54 ± 0.05^a^	0.3 ± 0.02^b^	0.53 ± 0.04^a^	0.26 ± 0.02^b^
Pb DTPA (mg kg^−1^)	3.7 ± 0.2^a^	1.3 ± 0.1^b^	3.3 ± 0.26^a^	1.2 ± 0.15^b^
Ni-DTPA (mg kg^−1^)	2.3 ± 0.3^a^	0.98 ± 0.03^b^	2.9 ± 0.25^a^	1.1 ± 0.02^b^
Total Zn (mg kg^−1^)	166.1 ± 6.8^a^	63.3 ± 1.5^b^	164.3 ± 4.2^a^	62.6 ± 1.1^b^
Total Cu (mg kg^−1^)	55.7 ± 1.8^a^	45.1 ± 1.3^b^	55.6 ± 1.4^a^	45.2 ± 0.98^b^
Total Cd (mg kg^−1^)	5.3 ± 0.97^a^	1.9 ± 0.3^b^	5.2 ± 1.3^a^	1.8 ± 0.25^b^
Total Pb (mg kg^−1^)	80.4 ± 2.5^a^	46.4 ± 1.2^b^	66.1 ± 2.1^a^	33.6 ± 0.94^b^
Total Ni (mg kg^−1^)	61.3 ± 3.2^a^	30.8 ± 1.1^b^	56.4 ± 1.9^a^	30.7 ± 0.97^b^

SD: standard deviation; LAS: leachate-affected soil; OM: organic matter; CCE: calcium carbonate equivalent; CEC: cation exchange capacity; EC: electrical conductivity; SAR: sodium adsorption ratio; ESP: exchangeable sodium percentage.

Different letters in each row show significant differences at P < 0.05 based on the paired t-test.

### Soil heavy metals

The concentration of bioavailable fraction, extracted by DTPA, of Zn, Cu, Cd, Pb, and Ni in LAS were estimated to be 15–49, 4–13, 0.2–1.1, 2.1–5.2, and 1.3–3.9 mg kg^−1^, respectively, while their content was 1.4–7.1, 1.5–5.5, 0.11–0.44, 0.6–2.3, and 0.4–1.7 mg kg^−1^ in the control soil, respectively (Table 2). The DTPA-fractions of all heavy metals were significantly higher in LAS than in the control soils. They were in the order of Zn > Pb > Ni > Cd > Cu. The same trend occurred at all soil sites, reflecting that the process of enriching the DTPA extractable-metals was promoted by leachate in the study area. In this context, Zn and Cd concentrations were several times higher than those found by other authors^29–31^ in soils similar to the study site (calcareous-semiarid soils). Therefore, soils influenced by leachate may not be advisable for crop production given DTPA-Zn and Cd as reported in previous works^3,32^. Safety and phytotoxic problems caused by the available fraction of these two elements have also been reported in previous works. The possible reason for the increase in Zn and Cd may be the high amount of residual vehicle tires, batteries, colored materials, and colored glass and inks on paper that are commonly found in the waste^6,33^. Similar to available fraction, the total fraction of five heavy metals was significantly conditioned by leachate. Enriched values of metals were found to be in the order of Cd (180–200%) > Zn (155–164%) > Ni (84–99%) > Pb (62–100%) > Cu (23–32%). However, the mean values of the metals were lower than their acceptable ranges except for Cd. This implies that the total Cd contamination (as with its available fraction) occurred when leachate entered the soil. Soil pollution by Cd-induced leachate has also been found in other works, e.g., Liu et al.^34^ from China and Adamcová et al.^35^ from the Czech Republic. The values of heavy metals and other soil variables varied depending on the soil site, which might have contributed to the properties of the leachate itself (e.g., the quantity and quality of the leachate) and the impact of the leachate on the affected soils.

### Soil Quality Indices (SQI)

#### The TDS approach

Given twenty-eight soil variables, the SQI was calculated using the TDS approach and two IQI and NQI models (IQI-T and NQI-T). Considering the communality analysis (Table 3), the indices of soil salinity-sodicity (e.g., EC, SAR, and ESP) and soil heavy metal (e.g. Zn, Cu, and Cd) had the highest weight (ranging from 0.04 to 0.042), suggesting that these variables can have a remarkable impact on the IQI-T and NQI-T. The importance of these variables on TDS can be attributed to their role in multiple soil functions and processes that are important from the soil quality degradation aspect^36^. In the LAS, a range from 0.44 to 0.6 with a mean value of 0.47 and from 0.34 to 0.39 with a mean value of 0.36 were recorded for the IQI-T and NQI-T, respectively.

**Table 3:** The communality and weight values of each soil variable for both TDS and MDS approach.

	TDS methods		MDS methods	
	Commnuity	Weight	Commnuity	Weight
pH	0.638	0.026		
EC (dS m^−1^)	0.998	0.042	0.943	0.208
SAR	0.992	0.041	0.934	0.204
ESP(%)	0.990	0.040		
OM (g kg^−1^)	0.979	0.040	0.916	0.20
CEC (cmol kg^−1^)	0.939	0.038		
CCE (g kg^−1^)	0.738	0.030		
Total N (%)	0.948	0.039		
Available P (mg kg^−1^)	0.781	0.032		
Available K (mg kg^−1^)	0.726	0.030		
Exchangeable Ca (cmol_c_ kg^−1^)	0.845	0.034	0.829	0.181
Exchangeable Mg (cmol_c_ kg^−1^)	0.858	0.035		
Exchangeable K (cmol_c_ kg^−1^)	0.924	0.038		
Exchangeable Na (cmol_c_ kg^−1^)	0.922	0.038		
Soluble Ca (meq l^−1^)	0.870	0.036		
Soluble Mg (meq l^−1^)	0.938	0.039		
Soluble K (meq l^−1^)	0.893	0.035		
Soluble Na (meq l^−1^)	0.908	0.037		
Zn-DTPA (mg kg^−1^)	0.993	0.041		
Cu- DTPA (mg kg^−1^)	0.979	0.040		
Cd-DTPA (mg kg^−1^)	0.948	0.041		
Pb DTPA (mg kg^−1^)	0.939	0.039		
Ni-DTPA (mg kg^−1^)	0.931	0.038		
Total Zn (mg kg^−1^)	0.935	0.040	0.957	0.209
Total Cu (mg kg^−1^)	0.947	0.039		
Total Cd (mg kg^−1^)	0.778	0.032		
Total Pb (mg kg^−1^)	0.968	0.040		
Total Ni (mg kg^−1^)	0.935	0.038		

TDS: total data set; MDS: minimum data set; OM: organic matter; CCE: calcium carbonate equivalent; CEC: cation exchange capacity; EC: electrical conductivity; SAR: sodium adsorption ratio; ESP: exchangeable sodium percentage.

**Figure 2:**
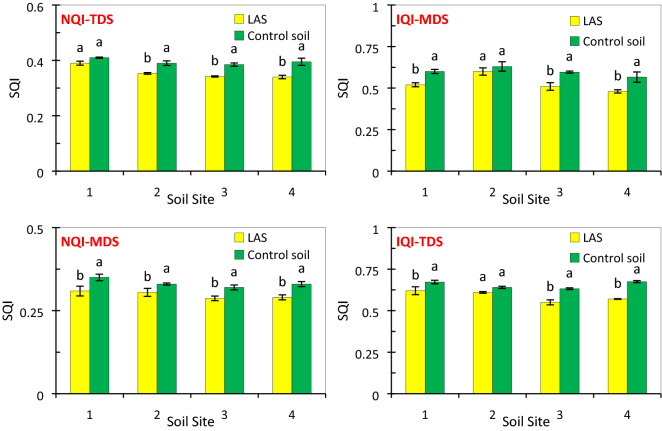
The comparison of the mean values of different soil quality index (SQI) scenarios between the leachate-affected soil (LAS) and the control soils.

The IQI-T and NQI-T were found in a range of 0.53 to 0.78 with a mean value of 0.62 and from 0.37 to 0.41 with a mean value of 0.39 in the control soils, respectively. The soil quality was grade III and grade IV in most soil sites affected by leachate and the control soils, respectively, regarding both IQI-T (0.52 > SQI ≥ 0.46) and NQI-T (0.37 > SQI ≥ 0.34) (Table 1). A considerable area of the grade III-IV soil quality has been reported in other studies in semi-arid regions^37–39^. Grade III has been identified as moderately suitable for plant growth but with some limitations while grade IV has more severe limitations for plant growth^26^. The IQT-T and NQI-T values dropped in the LAS, ranging from 5 to 16% and 6.5 to 13%, respectively, as compared to the control soils (Fig. 2) showing that the leachate has a negative and degradative effect on soil quality. The results corroborate the findings of other studies who found that municipal solid waste and landfill leachate cause the degradation of general soil quality by soil salinity and heavy metals^40–42^. The highest drop of IQT-T and NQI-T values were observed in site 4 where the highest rise occurred in the values of EC, SAR, and ESP for the LAS than those in the control. These variables contain the most important factors degrading soil quality in semi-arid environments (just like the current study), resulting in a greater decrease in SQI in site 4 than in the other sites. Soil salinity and sodicity indices (e.g., EC, SAR, and ESP) are also consistent with most indicators commonly used for soil quality assessment in arid and semi-arid areas^16,38,43^.

#### The MDS approach

As more research studies focused on quantifying SQI, it has become clear that its calculation is very costly and time-consuming if all soil attributes are used. Therefore, soil science researchers are trying to introduce methods that require fewer data while having good accuracy, which will, in turn, reduce the cost and time of SQI estimation. One of the most important alternatives is the minimum data set (MDS) method using principal component analysis, which is accepted by researchers and is widely applied^14,16,44^.

Here, a principal component analysis (PCA) was used to determine the MDS based on the standardized data matrix of the TDS (Table 4). Three PCs were specified to have eigenvalues > 1 ranging from 2.12 to 19.98, which accounted for 89.01% of the total variance in the original data. PC1, capturing 71.36% of the variance in data, had seven variables including EC, OM, available P, soluble K and Mg, and total Zn and Cu. There was a significant correlation (P < 0.01) between EC and soluble K and Mg, between OM and available P, and between total Zn and Cu. In the PC, EC, OM, and total Zn were retained in the MDS due to their higher-loading value. Referring to literature^8,12,16^, when the correlated variables in a PC are important factors affecting soil quality, those that include MDS are more important and the value of loading is higher. PC2 captured 10.06% of the total variance, and it was mostly loaded by SAR (0.959), ESP (0.911), exchangeable K (0.94), and Na (0.87), which were correlated with each other, and SAR was selected for the MDS. Under PC3, accounting for 7.58% of the total variance, exchangeable Ca and Mg were dominated with a loading value of 0.848 and 0.836, respectively.

**Table 4:** Data of principal component analysis (PCA) for soil quality variables.

PCs	PC1	PC2	PC3
Eigen value	19.98	2.82	2.12
Variance (%)	71.36	10.06	7.58
Cumulative variance (%) Eigenvectors	71.36	81.42	89.01
pH	− 0.730	− 0.274	0.174
EC	**0.968**	0.573	0.097
SAR	− 0.460	**0.959**	− 0.261
ESP	0.206	**0.911**	0.011
OM	**0.936**	0.577	0.234
CEC	− 0.672	0.503	0.477
CCE	0.777	0.239	− 0.298
Total N	− 0.768	0.514	0.308
Available P	**0.812**	0.238	0.253
Available K	0.273	0.55	− 0.591
Exchangeable Ca	− 0.070	0.259	**0.848**
Exchangeable Mg	0.351	− 0.192	**0.836**
Exchangeable K	0.200	**0.940**	− 0.034
Exchangeable Na	0.371	**0.870**	0.162
Soluble Ca	0.745	0.468	0.311
Soluble Mg	**0.834**	0.472	0.135
Soluble K	**0.901**	− 0.276	− 0.071
Soluble Na	0.695	0.651	− 0.034
Zn-DTPA	0.734	0.639	0.073
Cu-DTPA	0.727	0.591	0.263
Cd-DTPA	0.791	0.351	0.172
Pb-DTPA	0.759	0.622	0.07
Ni-DTPA	0.637	0.734	0.094
Total Zn	**0.979**	0.587	0.072
Total Cu	**0.813**	0.548	0.135
Total Cd	0.775	0.586	0.06
Total Pb	0.759	0.576	− 0.193
Total Ni	0.799	0.525	− 0.128

TDS: total data set; MDS: minimum data set; OM: organic matter; CCE: calcium carbonate equivalent; CEC: cation exchange capacity; EC: electrical conductivity; SAR: sodium adsorption ratio; ESP: exchangeable sodium percentage.

Underlined bold font values are considered highly weighted.

These two variables were significantly correlated with each other (P < 0.05) and exchangeable Ca (with more load value) was involved in the MDS. Consequently, five soil variables including EC, OM, total Zn, SAR, and exchangeable Ca were contained in the MDS in the present work. These findings followed the data of Andrews et al.^8^ and Karlen et al.^45^, who suggested that the quantitative soil quality assessment can be performed using at least five soil variables. The number of soil variables diminished from twenty-eight associated with the TDS to five in the MDS, showing a drop of more than 80% in soil variables using the MDS approach. This can significantly save money and improve work output. Besides, the soil variables included in the MDS of the study (mainly EC, OM, and ESP) are widely employed in the development of SQI by other researches, e.g., Nabiollahi et al.^38^ and Jahany and Rezapour^16^. This may be explained by the crucial effects of the variables on multiple soil functions including the combination of soil physical attributes, soil productivity, crop yield, and microbial community^6^. Data were found by De Clerck et al.^46^ suggesting that some soil variables (e.g. EC and organic matter) are most sensitive to management options and the most desirable of indicator to contribution into SQI. The equation for the integrated SQI using MDS (IQI-MDS) is given below:

$$IQI\text{-}MDS=0.209Zn+0.208EC+0.204SAR+0.20OM+0.181Ca$$

After leachate entered the soil, a range of 0.48–0.6 with a mean value of 0.53 and a range of 0.28–0.31 with a mean value of 0.30 was observed for the IQI-M and NQI-M, respectively. These indices (IQI-M and NQI-M) were recorded in a range of 0.56 to 0.63 with a mean value of 0.60 and 0.32–0.35 with a mean value of 0.33 in the control soils, respectively. Using the SQI grades of the MDS approach (Table 1), there were grades III and IV based on IQI (0.54 > SQI ≥ 0.58) and NQI (0.28 > SQI ≥ 0.31), respectively, in the majority of soil sites impacted by leachate which is almost similar to those determined for the TDS approach. On the other hand, the grades II and III soils were determined in the control sites using IQI-M and NQI-M, respectively, depicting that the grade of soil quality has dropped after adding the leachate to the soils, similar to what was occurred with the TDS approach. The SQI-MDS values ranged from 0.48 to 0.6 with a mean value of 0.53 for the IQI model and from 0.56 to 0.63 with a mean value of 0.6 for its control soil. For the NQI model, the values were in the range of 0.28–0.31 with a mean value of 0.3 and 0.32–0.35 with a mean value of 0.33 for its control soil. As with the TDS method, a drop of 5–15.2% and 7.5–12.2% was occurred in SQI using the IQI and NQI models (Fig. 2), implying that soil quality degradation was increased and stimulated by the leachate. The findings are in accordance with the results of previous studies that found that waste leachate can promote soil degradation by soil salinization and leaving organic and metal pollutants^47^.

Among the four soil sites, site 4 had the highest drop in both IQI-MDS and NQI-MDS followed by site 3, an almost similar tendency to the SQI-TDS approach. This may have resulted from the higher rate of the rise in EC and SAR after the leak of the leachate into the soil of sites 3 and 4 than the other sites. Given the weight of each variable (Table 3), the two variables are including the most powerful variables affecting MDS approach, resulting in more drop of SQI in some sites (e.g., sites 3 and 4) than other sites.EC and SAR have been widely used to describe SQI and have been specified to be very sensitive to agricultural management processes mainly in arid and semi-arid regions^6^. The degradative effects of EC and SAR may be linked to their negative impact on a combination of physicochemical and biological indicators of soil (e.g., soil structure, soil permeability and infiltration of water and air, soil osmotic pressure, soil fertility and nutrient cycling, and soil microbial community), thereby reducing plant growth, production, and vegetation covers^48^. The increasing pattern of EC and SAR in the LAS may be associated with the chemistry of waste leachate (Table S1) which is rich in the soluble salts (e.g., Ca^2+^, Na^+^, Cl^−^, and HCO_3_^−^), resulting in a significant increase in EC of the soils receiving leachate. On the other hand, the high content of Na^+^ and HCO_3_^−^ generated by leachate linked to the evapotranspiration process may lead to the depletion of Ca^2+^ and Mg^2+^ ions as insoluble carbonates (e.g., calcite and magnesite) whereas soluble Na is more concentrated in the solution and subsequently, it increases the SAR values in the LAS^16,48^.

### Relationship between SQI and wheat yield

**Figure 3:**
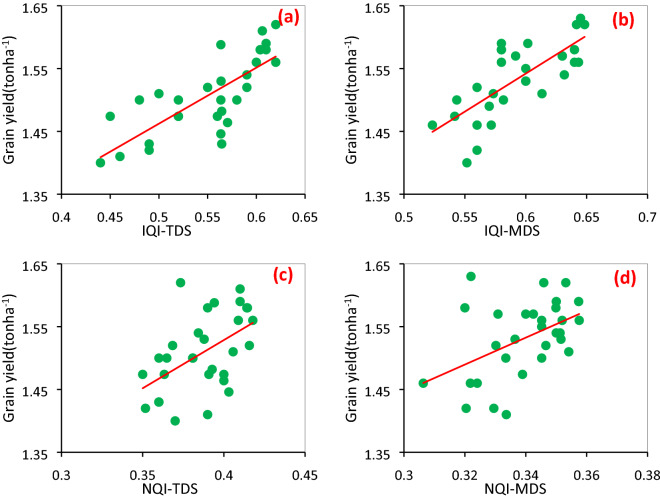
Wheat grain yield versus IQI-TDS (**a**), IQI-MDS (**b**), NQI-TDS (**c**), and NQI-MDS (**d**).

All SQI models (IQI-T, NQI-T, IQI-M, and NQI-M) had a significant relationship with the wheat yield based on the regression equations (Fig. 3), showing that the variations in the wheat yield could be captured by any of the models. The contributions of the wheat yield to SQI may be associated with the choice of soil variables (e.g., organic matter, macronutrients, and micronutrients), which may directly relate to crop performance. Several authors have suggested that crop yields are invariably associated with soil fertility indices and hence SQI may result in a high correlation with yields when some of the related attributes are involved in the SQI^14,49^. Our results are in line with several works from other regions, e.g., Vasu et al.^50^ from India, de Paul Obade and Lal^51^ from the US, and Li et al.^11^ from China who found the R^2^ of SQI versus crop yields in a range of 0.4–0.89.

The regression coefficients of IQI-T with the wheat yield was the highest, followed by IQI-M and NQI-T and NQI-M, implying that the IQI-T methods presented a better indication of variability in the wheat yield than the other methods. However, our suggestion to use the SQI-M for predicting wheat yield that its R^2^ has a narrow different with R^2^ of the IQI-T method because the TDS requires multiple numbers of soil indicators, increasing the time and cost of assessing soil quality. Moreover, in both LAS and controls soil, the mean value of sensitivity index (SI) was diminished in the order of IQI-M (1.35) > IQI-T (1.21) > NQI-M (1.15) > NQI-T (1.1), supporting more sensibility of the IQI-M model than the other models in assessing soil quality and wheat yield. Some researchers, including Mukhopadhyay et al.^52^, Raiesi^53^, and Karkaj et al.^54^, have compared different models of SQI evaluation by sensitivity index and found that the SI is an effective tool to assess the differentiating ability among different SQL scenarios. Using both TDS and MDS approaches, the regression coefficients of the IQI model were higher than those of the NQI model, reflecting that the IQI model provides a more accurate assessment of crop yield than the NQI model. This may be attributed to the fact that the combination of scoring and weighting is considered for soil variables in the IQI method whereas the NQI model is calculated only based on the average values and the minimum score of the characteristics^55^. Comparing different methods of determining SQI by Mukherjee and Lal^14^ showed that the IQI method had a stronger correlation with crop yield than the other methods due to the appropriate weight allocation for soil characteristics.

### Comparison of SQI models

All SQI models were significantly and positively correlated with one another with correlation coefficients ranging from 0.71 to 0.86 (Table 5), suggesting that either of the models could have been used to monitor soil quality in the present study. Nevertheless, the differentiating ability of SQI based on the IQI method was not the same as the SQI based on the NQI method.

**Table 5:** Correlation matrix for the eight SQI.

	IQI-T	IQI-M	NQI-T	NQI-M
IQI-T	1.00	0.88**	0.71**	0.81**
IQI-M	0.88**	1.00	0.74**	0.74**
NQI-T	0.71**	0.81**	1.00	0.71*
NQI-M	0.81**	0.74**	0.71*	1.00

**Correlation is significant at the 0.01 level.

*Correlation is significant at the 0.05 level.

In this respect, the correlation coefficients and SI (as discussed before) for IQI were always higher those for NQI had higher values. Comparing the different scenarios of SQI assessment, several researchers^38,44,55,56^ have found that the IQI method outperforms the NQI method in the assessment of soil quality.

Among the four models used in the present study, the IQI determined based on MDS was found to be the optimal model to estimate soil quality and predict crop yields given the analysis of the correlations among the SQI models, the correlations between the SQI models and wheat yield, and sensitivity index values.

## Conclusions

The present study compared 28 major chemical variables and SQI between waste leachate-affected soils and control soils using four models (e.g., IQI-TDS, IQI-MDS, NQI-TDS, and NQI-MDS) in an agricultural calcareous ecosystem. The leak of the leachate into soil increased the majority of soil variables mainly EC, SAR, ESP, total N, available P and K, exchangeable cations, and the available and total fractions of heavy metals significantly. Among the 28 variables included in the TDS approach, five soil variables (EC, OM, total Zn, SAR, and exchangeable Ca) were selected to the MDS approach using principal components analysis. This show a drop of more than 80% in total soil variables using the MDS approach, which can cut economic costs and improve work output. The contents of IQI and NQI were dropped by 5–16% and 6.5–13% for the TDS approach and by 5–15.2% and 7.5–12.2% for the MDS approach after the leak of the leachate into the soils. This implies that the leachate aggravates degradation and deterioration of soil quality. Therefore, new strategies and techniques are required to design appropriate management practices for leachate collection and inhibition of its movement through the development of new dumping sites with a proper foundation. Based on the comparison and evaluation of the accuracy of the different IQI models applied in the study, the data suggested that the IQI-MDS is an optimal model to assess soil quality and predict crop yield. However, we believe that the data need to be evaluated in other regions and ecosystems because they may be site-specific.

## Supplementary Information

Supplementary Information.
