# Supplementary material for: Degradation of soil quality by the waste leachate in a Mediterranean semi-arid ecosystem
Source: Sci Rep. 2021 May 31;11:11390. doi: 10.1038/s41598-021-90699-1 (PMC8166821; doi:10.1038/s41598-021-90699-1)
Supplement: Supplementary file 1 — Supplementary Information. [file 41598_2021_90699_MOESM1_ESM.docx]

**Table S1.** Selected chemical composition of the investigated leachate.

| Parameter | Unit | Mean | SD |
| --- | --- | --- | --- |
| pH | - | 5.6 | 0.32 |
| EC | dSm^-1^ | 5.4 | 0.73 |
| OM | % | 20.9 | 3.6 |
| Ca^+2^ | mg l^-1^ | 664 | 34.5 |
| Mg^+2^ | mg l^-1^ | 560 | 32.3 |
| K^+^ | mg l^-1^ | 345 | 78.2 |
| Na^+^ | mg l^-1^ | 828 | 126.1 |
| Cl^-^ | mg l^-1^ | 1775 | 211.3 |
| HCO_3_^-^ | mg l^-1^ | 1830 | 222.5 |
| Zn | mg kg^-1^ | 60.4 | 7.1 |
| Cu | mg kg^-1^ | 5.7 | 0.42 |
| Cd | mg kg^-1^ | 0.83 | 0.14 |
| Pb | mg kg^-1^ | 8.1 | 1.6 |
| Ni | mg kg^-1^ | 6.3 | 0.91 |
